# Supplementary material for: Green urbanization
Source: PLoS One. 2021 Nov 29;16(11):e0260393. doi: 10.1371/journal.pone.0260393 (PMC8629302; doi:10.1371/journal.pone.0260393)
Supplement: Attachment — Submitted filename: PLoSONE GreenUrbanization Review.pdf [file pone.0260393.s001.pdf]

Review of "Green Urbanization" for *PLoS ONE*

This paper examines the relationship between energy & pollution, and economic productivity in urban vs. non-urban areas. The authors find that energy consumption and waste generation is decreasing with population size in a study of UK metro areas.

In accordance with *PLoS ONE* requirements, my evaluation for acceptance or rejection of this article does not consider impact or interest level of the question, but instead focus solely on quality and sufficiency of data, technique, and results. The authors follow accepted methodology and use minimally sufficient and accepted data sources, resulting in acceptable analysis. However, I find these results to just clear the minimum possible hurtle defined by the Journal, with simplistic modeling and results identifying only correlation between population size and energy/pollution data. I recommend considering for publication following additional depth of analysis, and clear statement of the limitations of the insights. My comments follow.

1) Framing: The authors are working in a field already analyzed through a well-known major work: Glaeser's Triumph of the City. Its reasonable to assume that readers of this study may be familiar with that book, as well as the Glaeser's (and others) larger body of academic research which delves deeply into questions posed in this study. Further, the book was published in 2011, availing it of data from a similar time period which the authors use (2009). Therefore, the authors should speak plainly about how their work adds to the conversation which the aforementioned literature covers more deeply and broadly. While this concern of marginal contribution is not considered in evaluation of the paper for acceptance in this Journal (per Journal standards), clarification of the contribution will strengthen the paper's story and its placement within the related literature.
    a. Policy Proposal: In the Conclusion the authors indicate that they are proposing a policy. This falls outside the accepted scope of the Journal's mandate, and I was unclear as to what policy they were referring. This language and inclusion should be revisited for compliance and clarity.
2) Data Questions:
    a. The authors indicate that they are interested in the effects of density, yet are measuring city size ("large cities") solely in terms of total population. It may prove insightful to evaluate based on population density, not just count, as cities consume differing amount of land, and therefore do not present consistent definitions of density.
    b. In the alignment efforts between TTWAs and LAs, the authors indicate that they lose 202 of their 380 studied cities, with that overwhelmingly excluding smaller cities. What are the selection bias implications of this, and how may they be shaping your results?
    c. Waste analysis reveals rather notable structural breaks between residential vs. non-residential and city vs. non-city creation of waste and recycling. As waste and recycling are managed by the LAs, are there program supply implications to be considered here? Are these programs priced the same for consumers in the different categories? Are recycling programs available with equal depth and ease of access to the different consumer groups, and in different geographic areas? How do these aspects color the findings regarding waste?
    d. Are the measures of energy consumption primary only or total?

3) Methodology Questions:
   a. Are the presented result effects (for example, productivity gain of 7.2% per worker) linear? How do we interpret this marginal impact with respect to possible structural breaks? Perhaps an analysis of these findings at a 1 or 2 standard deviation point will add to the discussion.
   b. Data evaluating economic impacts are from 2009. Is this the best timeframe to use given coincidence of the Global Financial Crisis? If this is the only data available, the possible implications should be discussed.
   c. The analysis presented speaks only to the correlation with total population, rather than any causal impacts. The authors should speak to this in the text when describing the results, and should also consider ways in which they could identify causal relationships between density and consumption. This is particularly key in their discussions regarding people moving to cities.
   d. The authors model the location decision solely in terms of wages and house prices. They identify that these are endogenous, which will need addressed in the creation of their model and results discussion. However, this is also an oversimplification of the decision. Other examples of factors which shape the decision include school quality, commute time, and tax implications. If the authors wish to use this simplified version of the model, they must speak to its limitations in-text.
      i. With respect to tax implications, the authors specify that people living in cities do not pay a higher tax rate. However, this is not the case in many places. Often, cities are subject to notably higher tax rates across several categories, including higher land transfer tax rates, higher income tax rates, etc.
   e. The authors model the decision to move (assumedly domestically) from small cities to larger cities, but what about the reverse? International immigration overwhelmingly places people in large cities, but one of the largest sources of population loss from large cities is domestic out-migration from large cities to smaller cities. How is this accounted for in the author's model and story?
4) Additional comments:
   a. This paper is in need of thorough copyediting.
   b. Table 1 is unnecessary.
   c. The cities listed on Figures 6 and 7 are unreadable.
   d. I do not understand why the paper order is: Introduction; Results; Data and Methods; Conclusion. Please identify your data and methodology prior to presenting results so the reader has the context needed to evaluate the results.
