# Supplementary material for: Green urbanization
Source: PLoS One. 2021 Nov 29;16(11):e0260393. doi: 10.1371/journal.pone.0260393 (PMC8629302; doi:10.1371/journal.pone.0260393)
Supplement: Attachment — Submitted filename: reply_referees.pdf [file pone.0260393.s003.pdf]

Reply to Referees

Referee 1 – report titled "Review of "Green Urbanization" for PLoS ONE"

1) Framing: The authors are working in a field already analyzed through a well-known major work: Glaeser's Triumph of the City. Its reasonable to assume that readers of this study may be familiar with that book, as well as the Glaeser's (and others) larger body of academic research which delves deeply into questions posed in this study. Further, the book was published in 2011, availing it of data from a similar time period which the authors use (2009). Therefore, the authors should speak plainly about how their work adds to the conversation which the aforementioned literature covers more deeply and broadly. While this concern of marginal contribution is not considered in evaluation of the paper for acceptance in this Journal (per Journal standards), clarification of the contribution will strengthen the paper's story and its placement within the related literature. a. Policy Proposal: In the Conclusion the authors indicate that they are proposing a policy. This falls outside the accepted scope of the Journal's mandate, and I was unclear as to what policy they were referring. This language and inclusion should be revisited for compliance and clarity.

Thank you for your suggestion regarding the framing. We now include the discussion that you suggest.

We have changed the language "policy proposal" and now talk about a counterfactual in the context of the model that predicts what happens under different tax systems.

2) Data Questions: a. The authors indicate that they are interested in the effects of density, yet are measuring city size ("large cities") solely in terms of total population. It may prove insightful to evaluate based on population density, not just count, as cities consume differing amount of land, and therefore do not present consistent definitions of density.

Thank you for this suggestion. We now have also done the analysis for density, reported just below. Because density and size are highly correlated (see in the Figure below), the results using density are very similar.

The main result regarding the energy premium is virtually unchanged when using density instead of the wage bill as a regressor. The implied urban energy premium (energy usage per unit of output) is 18% according to the regressions using density as a regressor, while the main reported results is 17% (see Table 1).

Table 1. Energy Premium and Density

Dependent Variable	Log Wage Bill	Log total energy
OLS Coefficient - log density	1.084	0.902
S.E.	(0.046)	(0.042)

b. In the alignment efforts between TTWAs and LAs, the authors indicate that they lose 202 of their 380 studied cities, with that overwhelmingly excluding smaller cities. What are the selection bias implications of this, and how may they be shaping your results?

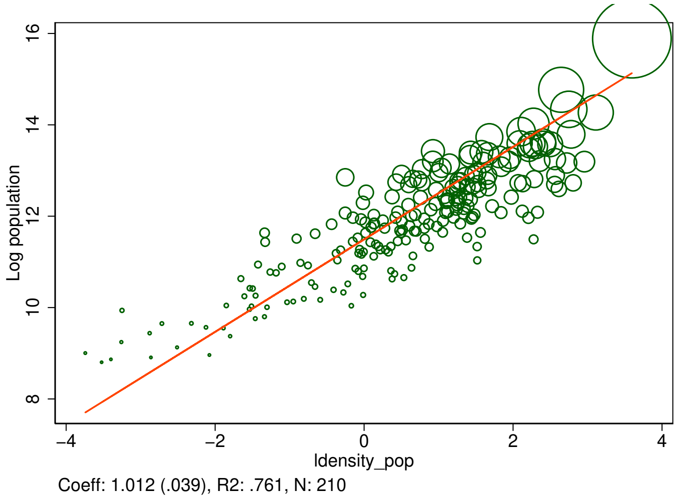

Fig 1. Density vs size

Thank you for raising this issue. We were imprecise in our description. We lost 202 LAs, not cities. Small LAs are common in both small and large cities (e.g. city of London as a LA has a small resident population, but missing it would have a negligible effect on estimated wages/population of London). Most importantly, we do not lose many TTWAs. We have now 210 of 218 TTWAs. We have updated the data description in the text. The data preparation was also updated to TTWA definitions of 2011 in order to provide results for more recent years, we now refer to 2010 in the body of the paper and provide additional results for other years below.

c. Waste analysis reveals rather notable structural breaks between residential vs. non-residential and city vs. non-city creation of waste and recycling. As waste and recycling are managed by the LAs, are there program supply implications to be considered here? Are these programs priced the same for consumers in the different categories? Are recycling programs available with equal depth and ease of access to the different consumer groups, and in different geographic areas? How do these aspects color the findings regarding waste?

This is an interesting point. This opens the debate on why residential and non-residential waste differs. That said, our analysis is based on the outcomes of waste and energy, irrespective of the motives. There will be many reasons why there are differences across different locations. Of course, in a world with competitive markets, prices for waste/recycling services should not differ. And citizens choose their representation in the political economy to optimize the waste management of the local community. All that said, we focus here on the measurement of the outcome and remain agnostic about the important reasons for differences that you point out.

d. Are the measures of energy consumption primary only or total?

The measures for energy consumption are total. This link for the data/methods description confirms this: https://www.gov.uk/government/collections/total-final-energy-consumption-at-sub-national-level

We have updated the data description.

3) Methodology Questions: a. Are the presented result effects (for example, productivity gain of 7.2% per worker) linear? How do we interpret this marginal impact

with respect to possible structural breaks? Perhaps an analysis of these findings at a 1 or 2 standard deviation point will add to the discussion.

Thank you for this suggestion. We have done further analysis to elucidate how the elasticity changes in the distribution. We have estimated the scaling coefficient separately for cities below and above median size. We find that the difference is generally insignificant. In the next Table, we report the difference in the scaling coefficient between the cities above and those below the median size.

Table 2. Difference in scaling coefficient wrt wage bill among cities below vs. above median population

	Scaling Coeff Diff	SE	Lower CI - 95%	Upper CI - 95%
Log total energy	-0.086	0.088	-0.258	0.085
Log household energy	0.009	0.057	-0.103	0.120
Log transport energy	-0.258	0.076	-0.408	-0.108
Log industrial energy	-0.026	0.15	-0.32	0.267

We find that the estimated scaling coefficients for total energy usage, household energy usage and industrial energy usage are not distinguishable statistically for small vs large cities. However, transport energy demand shows different scaling among smaller vs larger cities. This is consistent with some forms of public transport (like an underground train system) needing a minimal size to operate efficiently.

When repeating the same exercise for the scaling of the wage bill with respect to population size of cities, we find that the difference in the scaling coefficient is estimated at 0.092 (S.E. 0.065). So the null hypothesis, that productivity gains of working in a larger city are the same across the city size distribution, is not rejected at the 95% level.

b. Data evaluating economic impacts are from 2009. Is this the best time frame to use given coincidence of the Global Financial Crisis? If this is the only data available, the possible implications should be discussed.

This is potentially an important point. However, we find that the result for the scaling of total energy usage with respect to the wage bill is remarkably stable over time (see next Table). While total energy usage has changed over the same period, the relative efficiency of small and large cities remains almost identical.

Table 3. Estimated scaling coefficient of total energy wrt wage bill

Year	coeff	SE
2010	0.837	0.012
2011	0.831	0.013
2012	0.832	0.013
2013	0.832	0.013
2014	0.824	0.012
2015	0.822	0.012
2016	0.818	0.012

c. The analysis presented speaks only to the correlation with total population, rather than any causal impacts. The authors should speak to this in the text when describing the results, and should also consider ways in which they could identify causal relationships between density and consumption. This is particularly key in their discussions regarding people moving to cities.

That is an important observation, thanks. While in our theory and the counterfactual there is a causal relation between populations size (or density) and energy use, we do not have any explicit analysis of the empirical causal relation, which we now make clear in the text. Note also that in the model setting, the causal relation is from city characteristics to energy use and not from individuals.

d. The authors model the location decision solely in terms of wages and house prices. They identify that these are endogenous, which will need addressed in the creation of their model and results discussion. However, this is also an oversimplification of the decision. Other examples of factors which shape the decision include school quality, commute time, and tax implications. If the authors wish to use this simplified version of the model, they must speak to its limitations in-text.

i. With respect to tax implications, the authors specify that people living in cities do not pay a higher tax rate. However, this is not the case in many places. Often, cities are subject to notably higher tax rates across several categories, including higher land transfer tax rates, higher income tax rates, etc.

This is a valid point, and indeed there are more factors than just labor income and housing prices that we do not highlight in the model. That said, the model does include other factors captured by the amenities, both positive and negative. They are measured as a residual. While one could interpret the amenities as the "measurement error" of the data relative to the prediction of the model, we prefer the interpretation that they measure differences across cities in the value of services and aspects of a city, whether those are positive (entertainment, culture, public transportation,...) or negative (congestion,...). Most importantly, independent empirical evidence suggests that directly measured amenities are not correlated with city size (see [1]). This is in line with amenities not being correlated with size in our analysis.

And you are certainly right that taxes are not merely levied on labor. However, labor income taxes are the major source of government income, therefore we consider the tax counterfactual considered to be of particular relevance.

e. The authors model the decision to move (assumedly domestically) from small cities to larger cities, but what about the reverse? International immigration overwhelmingly places people in large cities, but one of the largest sources of population loss from large cities is domestic out-migration from large cities to smaller cities. How is this accounted for in the author's model and story?

Thank you for raising this point. Our model has predictions for net migration. Gross migration flows are substantially larger, double to triple the net migration rate. Individuals and families have other reasons to move than the wage of the worker, such as preferences, joint location decisions within the family,... While we can model the gross migration with a richer set of stochastic shocks, the equilibrium allocation is fully determined by the arbitrage of the marginal person moving and who at equilibrium prices be indifferent between staying and moving. Additional heterogeneity will thus lead to gross migration and strict indifference between moving and staying, but the equilibrium prices will not change. And then the heterogeneity can even be correlated with different sorts of characteristics: international born, personal traits such as age, education,...

4) Additional comments: a. This paper is in need of thorough copyediting.

b. Table 1 is unnecessary.

c. The cities listed on Figures 6 and 7 are unreadable.

d. I do not understand why the paper order is: Introduction; Results; Data and Methods; Conclusion. Please identify your data and methodology prior to presenting results so the reader has the context needed to evaluate the results.

a. We have copyedited the paper.

b. We have cut Table 1 and have added the information to the Caption of Figure 2

c. We now have increased the font of the names of those cities

d. We have changed the order of the sections in the paper: 1. Introduction; 2.1 Data; 2.2 Empirical Results; 3.1 Model and 3.2 Tax Counterfactual;

Referee 2 – report starts with "This manuscript provided new evidence"

1. The motivation of your research is not clearly shown in the abstract. Some descriptions are not closely related not your topic.

2. In the Introduction, the authors need to further clearly state contributions and novelty in your paper.

3. The authors have reviewed many studies of green urbanization, but the research gap is not identified. Furthermore, the research motivation should be given with more details. It is suggested to review the related studies by contents, methods, and factors.

Thank you for your suggestion to improve the writing and the motivation of the paper. We have updated the abstract and the introduction explicitly spelling out the contribution of our research.

4. In the data section (Methods and Data), All data is for the 2009 cross-section; the author may need to update

This is a great suggestion. Unfortunately, the data for waste generation is not available for any later years. For energy use, we have repeated the analysis for multiple years and find no qualitative change in the results. See our response to Referee 1 above.

5. The contribution to this energy premium of the industry is 12%(Results). Experience knows that industry consumes the most energy. Can we find the conclusions of previous studies, verify and explain this result in detail?

Thank you for encouraging us to look at previous research on energy use in industry. We have not found any research that contradicts our data, that roughly about 38% of energy use can be attributed to business, one third to domestic use, and 29% to transportation (see caption to Figure 2). These numbers are directly from the UK energy consumption statistics. As far as we know, ours is the first study to calculate the elasticity that generates the energy premium.

6. In terms of the article structure, I think it's better to put the Methods and Data before the Results.

This is a great suggestion. We have changed the order. We now describe the data before the empirical results and the model setup before the tax counterfactual.

7. There are several repetitions between the conclusions and main contents, and it is recommended to summarize them with more refined sentences 8. In the conclusions and summarizing the actions taken and results, please strengthen the explanation of their significance. It is recommended to use quantitative reasoning comparing with appropriate benchmarks, especially those stemming from previous work.

Thank you for these suggestions. We have now rewritten the conclusion. We now summarize our findings using quantitative reasoning and highlight their significance.

9. The paper should be proofread for better readability and correction of any grammar issue before publication.

Thanks. We have corrected the mistakes and have proofread the paper.

Response to Referee 3 – Report titled "Review Reports"

1. Why use total wage bill rather than the city-level average wage to represent the productivity of a city? There are examples of the higher wages in a smaller city across countries if not within a country.

That is a valid point. Note that in our model, the productivity is indeed equal to the city-level average wage. There is a one-to-one relation between the wage bill and the wage rate given population. And indeed, there are smaller cities with higher wages. In our analysis these differences are absorbed by the amenities that are also, like the average wage, expressed in per worker terms. These differences are also taken into account in the counterfactual regarding the labor income tax.

2. The authors use total wage bill to represent total production, why not use city-level GDP, could the authors use city-level GDP to do a robustness check.

We agree that this is the obvious way to use city-level GDP. Unfortunately, we do not have the data on city-level GDP in comparable geographic units for the years of our waste and energy data. While we have updates for the energy data (see also our response to Referees 1 and 2), there are currently no updates for the waste data.

3. I think there is a mistake in Figure 2. The authors either use a wrong figure or get a wrong writing, should replace "log population" with "log wage bill"

Yes, thanks for pointing this out. We have corrected it.

4. Please correct some spelling mistakes.

Thanks. We have corrected the mistakes and have proofread the paper.

5. The author should talk more about the potential drawbacks of large cities, such as health effect of pollutants concentration, which also has an effect on the citizen's choice of city to live.

That is an important point. We have added a discussion on the negative effects of living in large, dense cities.

References

1. Albouy D. Are Big Cities Bad Places to Live? Estimating Quality of Life across Metropolitan Areas; 2011.
