# Supplementary material for: Green urbanization
Source: PLoS One. 2021 Nov 29;16(11):e0260393. doi: 10.1371/journal.pone.0260393 (PMC8629302; doi:10.1371/journal.pone.0260393)
Supplement: Attachment — Submitted filename: replytoreferees.pdf [file pone.0260393.s005.pdf]

Reply to Referees

Answers to Referee 1

Thank you for your comments, they are very helpful to improve the paper.

Major Comment

To clarify the possibility of endogeneity of unobserved factors that affect the results we have added a short paragraph. We highlight that the "residual factors" are potentially endogenous and that all results should be considered conditional on those. Futher, we give examples for such possible endogeneity.

Here the paragraph we added in the model section: *"For the analysis in this paper we take those unobserved differences as given, thus all results we present have to be considered as conditional on the noise term ε_i. However, theoretically is not entirely clear which direction those unobserved factors, if endogenous, would adjust. For example, it could be that negative externalities related to density, like increased travel times and worsening air quality dominate, or that higher density allows for improved amenities for entertainment. However, even if our results quantitatively do not take this into account, as long as citizens take those factors into account when making location decisions, the qualitative result is unchanged."*

Moreover, the evidence in the literature shows that total amenities are independent of city population [1]. That is, there are big variations in amenities, but those variations are not systematically related to the population of a city. This is consistent with our assumptions and, at the very least, does not contradict our approach.

To improve the presentation of the regression results, we first added the basic equation of the scaling relationship and state how the scaling coefficients are estimated. Further, we clarified the captions of the figures. They now state: *"The scaling coefficient was estimated by OLS and the standard error is shown in parentheses"* (or similar). The estimated scaling coefficient, its standard error and the R^2 of the regression are shown. To save on space we did not want to repeat the same information in a regression table.

Minor Comments

1. We have rewritten the statement to clarify that we are stating the question we attempt to answer. Therefore we also do not cite.
2. We have added citations.
3. We now use just population, instead of population size or size, throughout the whole paper to avoid confusion.

Word document comments

Thank you for your helpful comments. We have corrected the mistakes you have mentioned and checked through the remaining formulas for additional typos.

References

1. Albouy D. Are Big Cities Bad Places to Live? Estimating Quality of Life across Metropolitan Areas; 2011.
