# Supplementary material for: Green urbanization
Source: PLoS One. 2021 Nov 29;16(11):e0260393. doi: 10.1371/journal.pone.0260393 (PMC8629302; doi:10.1371/journal.pone.0260393)
Supplement: Attachment — Submitted filename: Review.docx [file pone.0260393.s002.docx]

Review Reports

The authors use various datasets to support the fact that large cities, not only more productive, but also more energy efficient and green. The paper is well written and well organized, and I think it could be accepted for publication after minor revision.

Comments:

1. Why use total wage bill rather than the city-level average wage to represent the productivity of a city? There are examples of the higher wages in a smaller city across countries if not within a country.

2. The authors use total wage bill to represent total production, why not use city-level GDP, could the authors use city-level GDP to do a robustness check.

3. I think there is a mistake in Figure 2. The authors either use a wrong figure or get a wrong writing, should replace “log population” with “log wage bill”

4. Please correct some spelling mistakes, for example:

5. The author should talk more about the potential drawbacks of large cities, such as health effect of pollutants concentration, which also has an effect on the citizen’s choice of city to live.
