# Supplementary material for: Green urbanization
Source: PLoS One. 2021 Nov 29;16(11):e0260393. doi: 10.1371/journal.pone.0260393 (PMC8629302; doi:10.1371/journal.pone.0260393)
Supplement: Attachment — Submitted filename: Review Reports.docx [file pone.0260393.s004.docx]

Review Reports

I am glad to see that the authors give good answers to most of my questions in this revised manuscript, but still have the one which hardly to improve due to data limitation.

I think the rest of the work that the authors need to do is to correct some tiny mistakes. For example,

$$p_{i}h_{i}=\alpha\tilde{\omega}_{i} instead of ph_{i}=\alpha\tilde{\omega}_{i} in the line 286$$

In Fig 5. (recycled vs. non-recycled) should not the miss the “)” , etc.
